# Plasma levels of TNF-α, IL-6, IFN-γ, IL-12, IL-17, IL-22, and IL-23 in achalasia, eosinophilic esophagitis (EoE), and gastroesophageal reflux disease (GERD)

**DOI:** 10.1186/s12876-019-0937-9

**Published:** 2019-02-11

**Authors:** Steven Clayton, Elliot Cauble, Ambuj Kumar, Nirav Patil, Dennis Ledford, Narasaiah Kolliputi, Maria F. Lopes-Virella, Donald Castell, Joel Richter

**Affiliations:** 1 Wake Forest Baptist Medical Center, Winston-Salem, NC USA; 2 University of South Florida, Division of Gastroenterology, Florida, USA; 3 Greenville Health System, Greenville, SC USA; 4 University of South Florida, Division of Allergy Immunology, Florida, USA; 5 Medical University of South Carolina, Division of Endocrinology, Charleston, SC USA; 6 Medical University of South Carolina, Division of Gastroenterology, Charleston, SC USA

**Keywords:** Achalasia, Eosinophilic esophagitis, GERD, Inflammatory cytokines, IL-6

## Abstract

**Abstract:**

An elevation of serum inflammatory biomarkers in achalasia patients compared with controls recently was demonstrated. It has not been determined whether the elevation of inflammatory cytokines is unique to achalasia or occurs with other diseases involving the esophagus. The primary aim of our study was to compare the differences in plasma immunological profiles (TNF- α receptor, IL-6, IFN-γ, IL-12, IL-17, IL-22, and IL-23) of patients with achalasia, eosinophilic esophagitis (EoE), and gastroesophageal reflux disease (GERD). A secondary aim of this study was to classify these same plasma cytokine profiles in the three achalasia subtypes.

**Methods:**

Plasma from 53 patients with achalasia, 22 with EoE, and 20 with GERD (symptoms plus esophagitis or + reflux study) were analyzed. Exclusion criteria: malignancy, autoimmune condition, immunodeficiency disorder, and treatment with steroids/immune modulating drugs. Cytokine levels were assayed via multiplex enzyme-linked immunosorbent assay (ELISA).

**Results:**

Our key finding revealed significant elevations in IL- 6 (*p* = 0.0158) in achalasia patients compared with EoE patients. Overall, plasma inflammatory biomarker patterns were not different in the three subtypes of achalasia.

**Conclusion:**

There were no differences between the cytokine levels of any of the measured biomarkers between the achalasia and GERD groups suggesting that luminal stasis does increase biomarker levels for any of the cytokines examined in our study. While these results are an early first step towards clarifying some aspects of the pathogenesis of achalasia, they bring about many more questions that require further investigation and expansion. Further investigation with a larger cohort and a broader panel of biomarkers is needed.

## Background

Achalasia is the quintessential example of esophageal dysmotility. Achalasia was first described by Sir Thomas Willis in 1674. He chronicled a patient with achalasia treated over a 15 year time period with sequential dilations performed using sponge button mounted on whalebone. Although physicians have recognized achalasia for over 350 years, many aspects of the disease pathogenesis remain unknown [22].

Achalasia is characterized by loss of ganglion cells in the esophagus and in the LES. The pathogenesis of achalasia possibly involves both an autoimmune and inflammatory responses, this could be triggered by viral infection in prone individuals [14].

Type1 T helper cells (Th1) produce interferon-gamma (IFN-γ) and tumor necrosis factor- alpha (TNF-α) which activate macrophages promoting cell-mediated immunity. Type 2 helper cells (Th2) produce antibodies for protection from parasites. T helper 17 (Th17) responses control of infection. Uncontrolled Th1 and Th17 responses are involved in the pathogenesis of organ specific autoimmune disorders, in contrast with Th2 responses which are responsible for atopic disorders [7]. TNF-α, IL-12 and IFN-γ are associated with Th1 responses. Th17 responses are mediated by the cytokines IL-17, IL-22 and Il-23 [29].

A review of the literature suggests that TNF-α, IL-6, IFN-γ, IL-12, IL-17, IL-22, and IL-23 theoretically could contribute to the underlying etiology of achalasia. IL-17 is a key mediator in several autoimmune diseases by recruiting neutrophils and activating innate immune cells, promoting B-cell functions, and facilitating the production of pro-inflammatory cytokines. IL-17 and IL-22 concentrations in myenteric plexus esophageal tissue of achalasia patients are higher versus control [12, 13]. IL-23 contributes to autoimmune inflammatory disorders involving the gastrointestinal tract. IL-23 has been associated with Crohn’s disease, ulcerative colitis, and peptic ulcer disease [1, 19]. Elevated IFN-γ and low levels of TNF-α are seen in chagasic mega-esophagus, a process similar to achalasia, compared to controls [6].

In 2012, Cauble et al. demonstrated an elevation of serum inflammatory biomarkers in achalasia patients compared with controls suggesting the possibility that a systemic inflammatory response may contribute to the pathogenesis of achalasia. In their study, levels of interleukin-12 (IL-12) (*p* = .031) and TNF-α (*p* = .026) were significantly elevated in the achalasia group compared to the controls. Also, there was a trend toward significance in interleukin- 6 (IL-6) levels in the achalasia group, but this difference did not reach statistical significance (*p* = .053) [5]. It is not known whether this elevation of biomarkers is unique to achalasia or occurs with other diseases involving the esophagus. Multiple studies have shown distinct cytokine profiles in tissue specimens in gastroesophageal reflux disease (GERD) patients, and the response is currently understood to be localized and not systemic [8]. In contrast, eosinophilic esophagitis (EoE) is considered to be a Type 2 T-helper cell (Th-2) mediated systemic response [24].

The primary aim of our study was to compare the plasma immunological profiles of plasma of TNF-α, IL-6, IFN-γ, IL-12, IL-17, IL-22, and IL-23 in patients with achalasia, EoE, and GERD. A secondary aim of this study was to classify these same plasma cytokine profiles in the three achalasia subtypes.

## Methods

### Study design (IRB approval)

This study was an exploratory multi-institutional collaboration between the divisions of Gastroenterology and Allergy and Immunology at the University of South Florida (USF), Morsani College of Medicine and the Medical University of South Carolina (MUSC) Department of Medicine, Division of Endocrinology. All patients evaluated at the Joy McCann Culverhouse Center for Swallowing Disorders USF between January 2013 and October 2015 with one of the three diagnoses were asked to participate in the absence of exclusion criteria. Plasma samples were collected only at USF and sent to MUSC for analysis.

Plasma from patients with the diagnosis of achalasia based on high resolution impedance manometry (HRIM), EoE (persistent esophageal mucosal eosinophilia defined as greater than 15 eosinophils per high powered field not responding to proton pump inhibitors (PPIs) and patients with GERD (defined by symptoms and either erosive esophagitis or positive reflux testing) were analyzed at the MUSC Department of Medicine, Division of Endocrinology. Biomarker levels were assayed via multiplex enzyme-linked immunosorbent assay (ELISA) (R&D Systems Inc., Minneapolis, MN). Exclusion criteria included previously diagnosed malignancy, autoimmune condition, immunodeficiency disorder, Chagas disease, Hepatitis C, and current treatment with oral, intravenous, or inhaled corticosteroids or immune modulating drugs.

### High resolution esophageal impedance manometry protocol

HRIM tracings were reviewed by two investigators (SC and JR), and patients meeting diagnostic criteria for achalasia based on the Chicago Classification version 3.0 were identified and subtyped. Patients with technically inadequate HRIM studies were excluded.

HRIM studies were performed as per standard clinical protocol. A solid-state HRIM catheter consisting of 36 circumferential sensors spaced 1 cm apart (Medtronic Inc., Shoreview, MN) was placed transnasally with the patient in a seated position. After a landmark calibration phase, ten 5 ml water swallows spaced 20 to 30 s apart were completed. All tracings were reviewed using Manoview Analysis Software v3.0 (Medtronic Inc., Shoreview MN). The manometric patterns were classified as type I achalasia if there was an elevated median integrated relaxation pressure (IRP) (> 15 mmHg) and 100% failed peristalsis. Type II achalasia was defined by an elevated median IRP (> 15 mmHg), failed peristalsis, and panesophageal pressurization in ≥20% of swallows. Type III achalasia was characterized by an elevated median IRP (> 15 mmHg), no normal peristalsis, and ≥ 20% premature contractions, defined as DL < 4.5 in accordance with Chicago classification guidelines version 3 [17].

### Plasma collection and cytokine analysis

Following a detailed explanation of the study a written informed consent was obtained from all participants. We obtained a blood sample to measure the biomarkers. Two tubes of blood per subject were obtained by the research coordinator using conventional venipuncture. Blood collected for serum was placed serum separator tubes (SST) red and grey tiger top tubes ±1% *v*/v (~ 100uL) Protease inhibitor cocktail (Sigma). Each sample was inverted 5 times. Collected specimens were kept upright at room temperature for 10 min then placed in an ice bucket to cool at 4 °C. All sera was sent for immediate processing with all samples included in the study being processed in under 1 h after collection. The samples were centrifuged for 9 min at 3000RPM at a temperature of 4°, and the plasma was separated into 100 μl aliquots placed in 0.5 ml cryrovials. The tubes were immediately stored in a secured freezer at − 70° Celsius. No hemolyzed specimens were included in the data of this study. All plasma samples were collected at the University of South Florida. Samples were analyzed for the following biomarkers:

- Cytokines: IL-6, IL-12 p70 subunit), IL-17, IL-22, IL-23, INF-γ and TNF-α.

Frozen samples were shipped overnight on dry ice to the MUSC Department of Medicine, Division of Endocrinology for analysis.

Once the specimens were ready for analysis, the aliquots were thawed on ice. The specimens were analyzed using Quantikine Human Immunoassays (R&D Systems Inc., Minneapolis, MN). Each kit varies in sample amount and/or dilution, assay incubation times, and volume of conjugate and substrate. Therefore, the assay procedures were performed according to manufacturers’ guidelines for all of the analyzed cytokines and receptors. All plates were read and calculated (after final incubation) on a Molecular Devices VERSAmax Microplate Reader. All immunoenzymatic assays were performed in duplicate.

### GERD diagnosis

The diagnosis of GERD was based on endoscopic evidence of erosive esophagitis based on the Los Angeles classification of erosive esophagitis and/or an abnormal combined multichannel intraluminal impedance and pH test off of all acid suppressive medications for seven days [23]. We included 20 patients in the study with GERD.

Combined multichannel intraluminal impedance (MII) and pH testing was performed using an ambulatory combined impedance-pH catheter (Medtronic, Fridley, MN). Following a fasting period ≥5 h, patients underwent esophageal manometry for the localization of the lower esophageal sphincter (LES) and assessment of esophageal function. The combined impedance-pH catheter was subsequently placed in the esophageal body with a reference pH electrode distal to the LES and two pH monitoring electrodes 5 cm and 20 cm proximal to the LES. The device also contained 8 impedance rings located at 2, 4, 6, 8, 10, 14, 16, and 18 cm proximal to the LES. The study catheter was attached to an external ambulatory monitor and worn for 24 h during which the study subjects were encouraged to maintain their normal activities, sleep schedules, and eating habits. In addition to impedance and pH data, the 24-h Imp-pH monitor provided information regarding changes in body position and the timing of meals and GERD symptoms, as noted by the patient. Tests not lasting longer than 21 h were excluded from data analysis.

After the study period, data from each monitor was transferred to a computer and visually analyzed using a commercially available software program (Given Imaging, Duluth, GA). Meals were marked and excluded from reflux analysis. Esophageal acid exposure time (EAET) was quantified as the percentage of time that the pH detected by the distal pH probe was below 4.0, or EAET.

The original pH definitions set by Johnson and Demeester (1974) were used as the standards for the detection of pathological levels of reflux events. Thus, > 4.2% total EAET, > 6.3% EAET in the upright position, and > 1.2% EAET in the recumbent position were the chosen standards [16].

### EoE

The diagnosis of EoE was made in patients with symptoms related to esophageal dysfunction (dysphagia, upper abdominal pain, chest discomfort, and reflux) and esophageal biopsies from both the proximal and distal esophagus revealing > 15 eosinophils/high powered field (hpf), without an alternative cause of eosinophilia. The persistence of esophageal mucosal eosinophilia on repeat esophageal biopsy after an adequate trial (8 weeks) of twice daily PPI therapy was required to be included in the study.

### Statistical analysis

The plasma immunological profile of IL-6, IL-12p70, IL-17, IL-22, IL-23, TNFα and INF-γ values obtained from patients with achalasia, EoE, and GERD was analyzed. The distribution of the data was assessed using the Kolmogorov-Smirnov test, and this analysis revealed that none of the biomarkers were normally distributed. Therefore, nonparametric statistical methods were used for further analysis. Median and interquartile range were used to describe each biomarker. The Kruskal–Wallis rank sum test was used to compare the composite scores among three groups. The *p*-value of less than 0.05 was considered to be significant. All the analyses were performed using SAS Enterprise Guide 7.1, (SAS institute, Cary, NC).

## Results

### Demographic data

The study included 53 patients with achalasia (mean age 61 years old (yo), 27 male**,** 26 female), 22 (mean age 40 yo, 15 male, 7 female) with EoE, and 20 (mean age 55, 11 male, 9 female) with GERD. In the GERD cohort, 16 patients had abnormal pH impedance testing and 4 patients had erosive esophagitis on endoscopy.

### Immunological profiles among patients with achalasia, EoE, and GERD

Table 1 summarizes the plasma biomarker median levels of IL-6, IL-12p70, IL-17, IL-22, IL-23, and TNF-α and INF-γ among the patients studied with achalasia, EoE, and GERD. The statistical comparisons between the different biomarker profiles of patients with achalasia, EoE, and GERD revealed statistically significant increases in IL-6 (p 0.05) in the achalasia group vs. EoE group. There were no significant differences between biomarkers in achalasia vs GERD.

**Table 1:** Comparison of biomarker levels among patients with achalasia, EoE, and GERD

Biomarker	Achalasia (*n* = 53)	EoE (*n* = 22)	GERD (*n* = 21)	*P*-value^a^
	Median (IQR)	Median (IQR)	Median (IQR)	
IL-6	2.45 (2.4)	1.71 (1)	2.05 (3.7)	0.0158 Achalasia vs EoE < 0.05^b^
IL-12p70	1.45 (0.9)	2.35 (1.4)	1.19 (0.98)	0.091
IL-17	8.14 (5.6)	9.96 (7.9)	5.89 (6.9)	0.052
IL-22	12.4 (5.7)	10.28 (3.3)	11.35 (4.2)	0.097
IL-23	23.86 (15.0)	26.58 (16.9)	16.45 (12.8)	0.10
TNF-α	4.06 (1.15)	3.97 (2.1)	4.17 (1.3)	0.53
IFN-γ	5.27 (1.7)	5.78 (1.5)	6.12 (1.9)	0.22

a - Kruskal–Wallis rank sum test to compare overall differences between groups

b - post-hoc Dunn’s test *p*-value for pairwise comparison

### Immunological profiles for the subtypes of achalasia

Of the 53 achalasia patient plasma specimens that were analyzed, 13(25%) were type I achalasia, 32(60%) were type II achalasia, and 8 (15%) were type III achalasia. The plasma biomarker median levels of IL-6, IL-12p70, IL-17, IL-22, IL-23, and TNF-α and INF-γ for achalasia subtypes 1, 2, and 3 are shown in Table 2. The *P* value calculated for these biomarkers in the three subtypes of achalasia did not show any statistical significance (*P* < 0.05) between groups. The study did not demonstrate the difference in plasma biomarker levels between the three subtypes of achalasia.

**Table 2:** Comparison of immunological profiles in the three subtypes of achalasia

Biomarker	Achalasia Type 1 (*n* = 13)	Achalasia Type 2 (*n* = 32)	Achalasia Type 3 (*n* = 8)	*P*-value^a^
	Median (IQR)	Median (IQR)	Median (IQR)	
IL-6	2.68 (1.8)	2.35 (2.7)	3.63 (2.4)	0.67
IL-12p70	1.44 (1.12)	1.5 (0.8)	1.6 (1.2)	0.79
IL-17	6.85 (6.3)	8.3 (5.6)	9.4 (3.4)	0.78
IL-22	12.8 (7.5)	11.8 (5.9)	13.6 (4.5)	0.47
IL-23	13.4 (14.7)	23.9 (13.5)	26.5 (11.1)	0.31
TNF-α	4.1 (0.8)	4.1 (1.3)	4 (1.6)	0.89
IFN-γ	5.6 (1.1)	5.6 (1.9)	4.5 (1.4)	0.29

a - Kruskal–Wallis rank sum test to compare overall differences between groups

Further, sub-analysis comparing the biomarkers the 3 achalasia subtypes to EoE and GERD did not demonstrate the any significant difference. Table 3. summarizes the analysis.

**Table 3:** Comparison of immunological profiles in the three subtypes of achalasia

Biomarker	Achalasia Type 1 (*n* = 13)	Achalasia Type 2 (*n* = 32)	Achalasia Type 3 (*n* = 8)	GERD (*n* = 21)	EoE (*n* = 22)	*P*-value^a^
	Median (IQR)	Median (IQR)	Median (IQR)	Median (IQR)	Median (IQR)	
IL-6	2.68 (2.16)	2.35 (2.40)	3.65 (2.53)	2.06 (4.56)	1.71 (1.02)	0.04
IL-12p70	1.44 (1.24)	1.43 (0.85)	1. 72 (1.95)	1.19 (1.38)	2.35 (1.48)	0.31
IL-17	6.85 (6.49)	8.28 (5.36)	98.93 (3.95)	5.9 (8.10)	9.96 (8.05)	0.25
IL-22	12.82 (9.14)	11.82 (6.14)	14.52 (5.33)	11.35 (6.28)	10.28 (3.53)	0.14
IL-23	13.45 (17.44)	23.88 (14.10)	25.64 (17.72)	16.45 (13.65)	26.58 (17.86)	0.18
TNF-α	4.06 (1.15)	4.03 (1.56)	4.16 (2.40)	4.12 (1.32)	3.97 (2.12)	0.9
IFN-γ	5.63 (1.21)	5.59 (2.33)	4.47 (0.94)	6.02 (2.05)	3.97 (2.12)	0.290.16

a - Kruskal–Wallis rank sum test to compare overall differences between groups

## Discussion

In this study, we investigated plasma biomarker levels of IL-6, IL-12p70, IL-17, IL-22, IL-23, TNFα and INF-γ in patients with achalasia, EoE, and GERD. Our key findings are:

- Significant elevations in IL- 6 in achalasia patients compared with EoE patients.
- Plasma inflammatory biomarker patterns were not different in the three subtypes of achalasia.

Although the etiology of achalasia remains unknown, there is increasing evidence implicating an inflammatory basis for the disease. In Caubles’ et al. original study comparing cytokine profiles in patients with achalasia versus healthy controls, IL-6 levels were elevated compared to health controls and closely approached but did not achieve statistical significance *p* = .052. In our study IL-6 levels did reach significance compared with EoE. These findings suggest that IL-6 is elevated in serum and plasma of patients with achalasia. IL-6 has been associated as part of the neuronal reaction to nerve damage and inflammation [20]. Ganglionitis of the myenteric plexus in achalasia patients leads to the destruction of the myenteric neurons that coordinate esophageal peristalsis and LES relaxation. Therefore, one would expect IL-6 levels to be elevated as a result of neuronal injury [4].

Elevated IL-6 levels, myositis, periganglionitis, and ganglionitis, as a result of high parasite load of *Trypanosoma (T.) cruzi,* the protozoan responsible for Chagas Disease, were described [27]. *T. cruzi* destroys the Meissner’s and Auerbach’s plexuses of the esophagus resulting in a clinical presentation similar to achalasia [28]. In both Chagas megaesophagus and achalasia, there is destruction of the neuronal plexuses. Both processes have been associated with increased IL-6 levels in the plasma, suggesting that elevated IL-6 levels may be indicative of myenteric plexus ganglionitis and neuronal apoptosis [21].

Elevated levels of IL-6 have been seen in other inflammatory conditions of the gastrointestinal tract, especially inflammatory bowel disease [21]. IL-6 triggers IL-21 production by human CD4 + T cells and IL-21 is an inducer of IL-22 production in CD4+ T cells [10, 13, 30, 31].

Typically, EoE has previously been characterized as a Th-2 type allergic immune mediated condition of the esophagus [26]. EoE results in persistent esophageal mucosal eosinophilia, defined as greater than 15 eosinophils per high powered field, without response to PPIs and symptoms of esophageal dysfunction [9]. EoE is associated with increased tissue levels of eotaxin-3 and IL-13 mRNA, suggesting a Th2-mediated inflammation and therefore IL-6 levels would not be expected to be elevated in the EoE patient population [2, 3, 18].

In Caubles’ et al. study, IL-12 levels were elevated in achalasia patients compared with health controls (*p* = 0.031) [5]. IL-12 induces development of Type-1 T helper cells (Th-1 cells), which produce INF-γ, and IL-23. IL-23 is involved in differentiation of Th17 cells in a pro-inflammatory context especially in the presence of TGF-β and IL-6. In our study, median IL-12 levels were higher in our EoE group compared to GERD and achalasia groups but did not reach statistical significance [11]. Active ganglionitis likely explains why the achalasia patients had significant elevations in IL- 6 compared with EoE patients.

The lack of differences in the cytokine levels of any of the measured biomarkers between the achalasia and GERD groups suggests that luminal stasis (vs neuronal inflammation) does not elevate any of the examined cytokines.

A secondary aim of this study was to classify the plasma biomarkers in the three achalasia subtypes. Impaired lower esophageal sphincter relaxation can occur in different achalasia subtypes but a disease-specific biomarker to differentiate the 3 subtypes has not been identified. Our study did not demonstrate a difference in plasma biomarker levels between the three achalasia subtypes.

The histopathologic features of 11 patients with achalasia compared to 8 esophagectomy controls were assessed by Goldblum et al. Inflammation was demonstrated histologically in all patients with achalasia but only the type I achalasia patients had evidence of neural fibrosis. This finding suggested a spectrum of histopathological changes at different stages of achalasia with persistent inflammation throughout the continuum of the disease [15].

Similarly, Sodikoff et al. studied the inflammatory infiltrate from LES muscularis propria biopsies at the time of laparoscopic myotomy. Lymphocytes were the predominant inflammatory cell in 7 out 8 cases (88%) with one case having an eosinophil-predominant infiltrate in the myenteric plexus. They found no difference in the proportion of inflammation demonstrated histologically between the different subtypes of achalasia. This suggested ongoing inflammation throughout the achalasia disease process [25]. Our findings support those of Goldblum et al. and Sodikoff et al, suggesting there is consistent cytokine release into the plasma across the three achalasia subtypes**,** indicating persistent inflammation throughout the clinical continuum of achalasia.

Some potential weaknesses of our study are: Plasma biomarkers levels may not accurately reflect tissue inflammation in one organ. Our sample size (*n* = 96) may have limited our ability to find associations. While significant time was spent deciding which specific biomarkers to study, our list of analyzed biomarkers is not at all inclusive. The findings of this study should prompt further investigation with a larger cohort and a broader panel of biomarkers. The aim of this study was to evaluate different biomarkers in achalasia and compare with other diseases involving the esophagus therefore we did not have a control group in our study which limits our study.

## Conclusions

Our results suggest that the inflammatory processes involved in achalasia, EoE**,** and GERD are distinct. There were significant elevations in IL- 6 in achalasia patients compared with EoE patients. However, the biomarkers profiles were not different in the three subtypes of achalasia. There were no differences between the cytokine levels of any of the measured biomarkers between the achalasia and GERD groups**,** suggesting that luminal stasis does increase biomarker levels for any of the cytokines examined in our study. While these results are an early first step towards clarifying some aspects of the pathogenesis of achalasia, they bring about many more questions that require further investigation and expansion. Further investigation with a larger cohort and a broader panel of biomarkers is needed.

## Abbreviations

EAET: Esophageal acid exposure
ELISA: Enzyme-linked immunosorbent assay
EoE: Eosinophilic esophagitis
GERD: Gastroesophageal reflux disease
hpf: High powered field
HRIM: High resolution impedance manometry
IFN-γ: Interferon –gamma
IL-12: Interleukin-12
IL-17: Interleukins-17
IL-22: Interleukin-22
IL-23: Interleukin-23
IL-6: Interleukin- 6
IRP: Integrated relaxation pressure
MUSC: Medical University of South Carolina
PPIs: Proton pump inhibitors
Th-1: Type 1 T-helper cell
Th-2: Type 2 T-helper cell
TNF-α: Tumor necrosis factor- alpha
USF: University of South Florida
yo: Years old
